# Supplementary material for: The interactome of intact mitochondria by cross-linking mass spectrometry provides evidence for coexisting respiratory supercomplexes
Source: Mol Cell Proteomics. 2017 Dec 8;17(2):216–32. doi: 10.1074/mcp.RA117.000470 (PMC5795388; doi:10.1074/mcp.RA117.000470)
Supplement: Supplemental Data [file supp_RA117.000470_133922_0_supp_23978_fzffwf.zip › spectra_annotation/mito_DR_spectra_annotation/455-1-6-1-1-1.pdf]

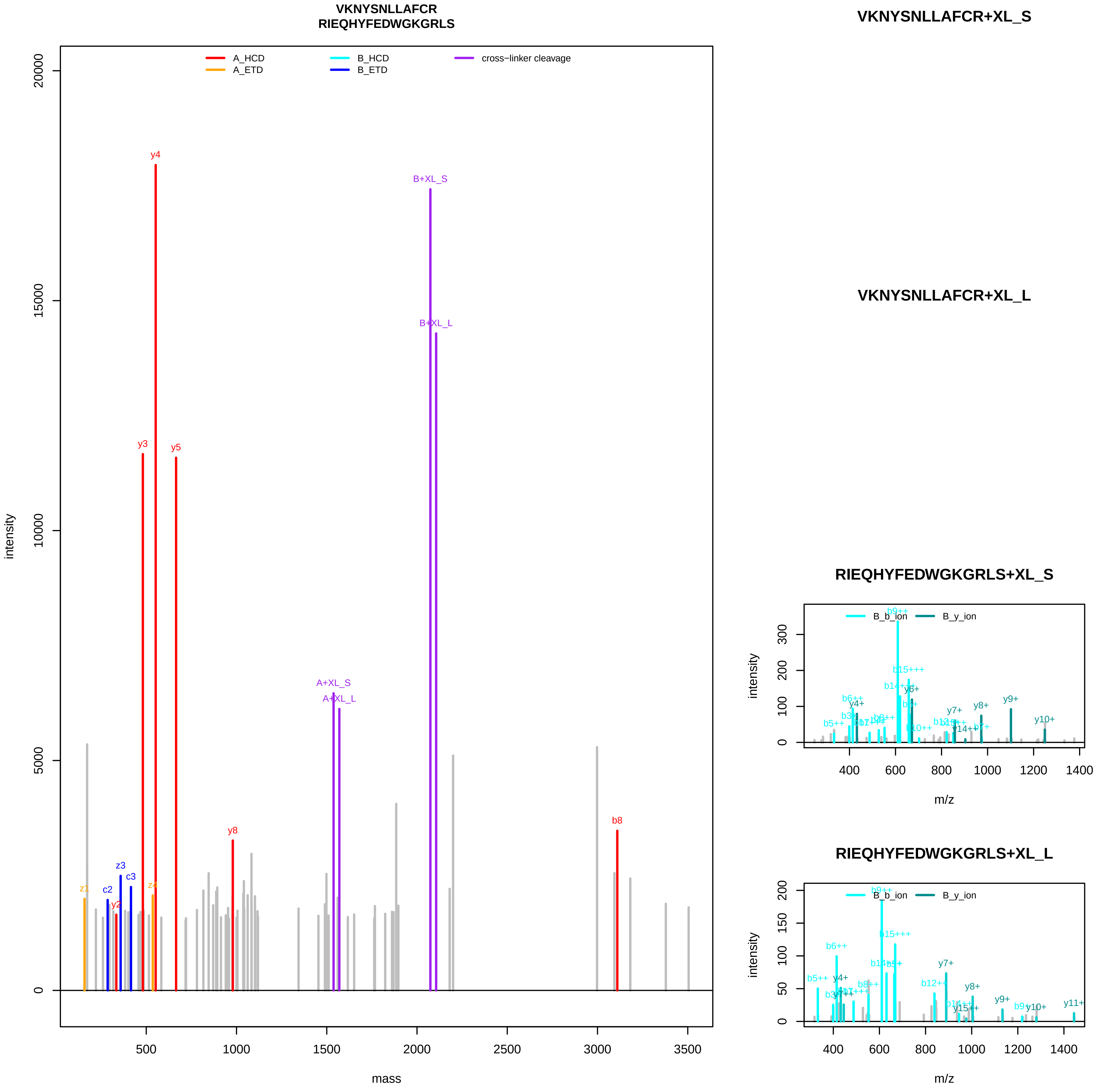
VKNYSNLLAFCR
RIEQHYFEDWGKGRLS
A_HCD
A_ETD
B_HCD
B_ETD
cross−linker cleavage
intensity
mass
0
5000
10000
15000
20000
500
1000
1500
2000
2500
3000
3500
y4
B+XL_S
B+XL_L
y3
y5
A+XL_S
A+XL_L
y8
b8
z1
c2
y2
z3
c3
z4
VKNYSNLLAFCR+XL_S
VKNYSNLLAFCR+XL_L
RIEQHYFEDWGKGRLS+XL_S
B_b_ion
B_y_ion
intensity
m/z
0
100
200
300
400
600
800
1000
1200
1400
b9++
b15+++
b14++
y6+
b6++
y4+
y9+
y8+
y7+
y10+
b5++
RIEQHYFEDWGKGRLS+XL_L
B_b_ion
B_y_ion
intensity
m/z
0
50
100
150
200
400
600
800
1000
1200
1400
b9++
b15+++
b6++
b14++
b5+
b7+
y8+
y9+
y10+
y11+
b12++
y4+
b5++
b8++
